# Perceval-S over time. Clinical outcomes after ten years of usage

**DOI:** 10.1186/s13019-024-02617-x

**Published:** 2024-04-09

**Authors:** Nikolaos Schizas, Ilias Samiotis, Georgia Nazou, Dimitrios C. Iliopoulos, Ioannis Anagnostopoulos, Maria Kousta, Nafsika Papaioannou, Mihalis Argiriou, Panagiotis Dedeilias

**Affiliations:** 1 Cardiovascular and Thoracic Surgery Department, Evangelismos General Hospital, Athens, Greece; 2 Department of Anesthesiology, Evangelismos General Hospital, Athens, Greece; 3 National and Kapodistrian University of Athens, Athens, Greece; 4 Department of Cardiology, G. Gennimatas General Hospital, Athens, Greece; 5 Environmental Engineering Laboratory, Aristotle University, Thessaloniki, Greece

**Keywords:** Sutureless aortic valves, Perceval-S, Aortic valve stenosis, Surgical AVR, Aortic valve replacement

## Abstract

**Background:**

Perceval-S has become a reliable and commonly used option in surgical aortic valve replacement (AVR) since its first implantation in humans 15 years ago. Despite the fact that this aortic valve has been proven efficient enough in the short and mid-term period, there is still lack of evidence for the long-term outcomes.

**Materials and methods:**

This is an observational retrospective study in a high-volume cardiovascular center. Pertinent data were collected for all the patients in whom Perceval-S was implanted from 2013 to 2020.

**Results:**

The total number of patients was 205 with a mean age 76.4 years. Mean survival time was 5.5 years (SE = 0.26). The overall survival probability of patients undergoing aortic valve replacement with Perceval-S at 6 months was 91.0% (Standard Error SE = 2.0%), at one year 88.4% (SE = 2.3%) and at 5-years 64.8% (SE = 4.4%). A detrimental cardiac event leading to death was the probable cause of death in 35 patients (55.6%). The initiation of Transcatheter Aortic Valve Replacement (TAVR) program in our center in 2017 was associated with a decline in the number of very high-risk patients treated with sutureless bioprosthesis. This fact is demonstrated by the significant shift towards lower surgical risk cases, as median Euroscore II was reduced from 5,550 in 2016 to 3,390 in 2020. Mini sternotomy was implemented in 79,5% of cases favoring less invasive approach. Low incidence of reinterventions, patient prosthesis mismatch and structural valve degeneration was detected.

**Conclusions:**

The survival rate after aortic valve replacement with implantation of Perceval-S is satisfactory in the long-term follow-up. Cases of bioprosthesis dysfunction were limited. Mini sternotomy was used in the majority of cases. TAVR initiation program impacted on the proportion of patients treated with Perceval-S with reduction of high-risk patients submitted to surgery.

## Introduction

After almost 15 years since its initial implantation in 2007 on human, Perceval-S constitutes one of the basic options for aortic valve replacement among many bioprotheses [1]. Since then more than 50,000 patients have been treated with this valve worldwide. This prosthesis is related with many important advantages including its ease of usage due to placement without the need of sutures on the aortic annulus, the significant reduction in procedural time, its compatibility with minimally invasive techniques and very satisfactory valvular function especially in the elderly with small aortic root [2, 3, 4]. Its design aims to increase effective orifice area of the aortic valve and its nitinol based stent improves elasticity and biocompatibility of the valve. Moreover, evidence from comparative studies showed that self-expandable rapid deployment bioprosthesis is a reliable alternative to traditional bioprosthetic valves. In a recent propensity score matched analysis was found that 30-day mortality, major comorbidities and paravalvular leakage were similar between traditional and rapid deployment valves, while rapid deployment valves were related to shorter operative times, smaller incisions and higher rates of pacemaker placement [5]. Even though the efficacy of Perceval-S has been extensively studied in the early and mid-term postoperative period, there is still lack of evidence for long term outcomes. More specifically, large randomized clinical trials, such as the PERSIST-AVR trial, are on progress nowadays 6. In this article, the whole experience of a single high-volume center, in which the first sutureless valve was implanted in 2013, is presented.

## Materials and methods

This is a retrospective observational study including all the patients receiving sutureless valve (Perceval-S) in a high-volume cardiovascular center since June 2013, when the first valve of this type was implanted, till December 2020. The choice of this type of valve was mostly made based on the patient’s background and especially when reduced operative time was crucial for the outcome. More specifically, patients with serious comorbidities and increased perioperative risk were the majority of the participants of the study. Data were collected from the hospital’s registration system and were crosschecked for the providers of the valve. The total number of the participants was 205. Information regarding the patients were assessed through annual follow-up examination including clinical and echocardiographic examination.

Prior to the commencement of the study, written approval of the ethics committee of the hospital was obtained (approval number 625.20/01/2022). The whole investigating and experimenting process was in accordance with the Declaration of Helsinki and the local legislation in Bioethics. The personal data and rights were protected according to the law.

### Statistical analysis

Normal distributed variables are expressed as mean (Standard Deviation) or as median (interquantile range). Qualitative variables were expressed as absolute and relative frequencies. Kruskall-Wallis test was used for the comparison of patients’ BSA among all four valve sizes. Spearman correlation coefficient was used to explore the association of BSA and valve size. Life table analyses were used to calculate cumulative survival rate (standard errors) for specific time intervals. The prognostic value of each variable was first assessed by univariate Cox regression analysis. Variables that showed significant association with the outcome were included in the multivariate Cox proportional-hazard model in a stepwise method in order to determine the independent predictors for surviving. Hazard ratios (HR) with their 95% confidence intervals were computed from the results of the Cox regression analyses. The assumption of proportional hazards was evaluated by testing for interaction with a continuous time variable. Kaplan – Meier survival curve was graphed over the follow-up period. All reported p values are two-tailed. Statistical significance was set at *p* < 0.05 and analyses were conducted using SPSS statistical software (version 22.0).

## Results

Sample consisted by 205 patients (65.9% females) with mean age 76.4 years (SD = 6.1 years). Their characteristics are presented in Table 1. Median BSA (Body Surface Area) was 1.76 (IQR: 1.66 ─ 1.91). The majority of the patients (66.8%) underwent isolated AVR. Mini sternotomy was performed only in isolated AVR cases with its percentage reaching 79,5%, versus 28 patients (21,5%) treated with full sternotomy. Mean valve size was 23.9 (Standard Deviation SD = 1.9) and in 41.0% of patients medium sized valve was used. The mean follow-up period was 6.7 (2.3–9.4) years. All patients included in the study had completed the follow-up.

**Table 1:** Sample characteristics

	Ν (%)
Sex	
Men	70 (34.1)
Women	135 (65.9)
Age, mean (SD)	76.4 (6.1)
BSA, median (IQR)	1.76 (1.66 ─ 1.91)
EUROSCORE II mean (SD)	4.101 (0.9–14.2)
EF (Ejection Fraction of Left Ventricle) %, mean (SD)	51.9 (10.3)
Valve size	
Small	29 (14.1)
Medium	84 (41.0)
Large	59 (28.8)
Extra large	33 (16.1)
Disease	
Aortic valve stenosis	185 (90.2)
Coronary artery disease	62 (30.2)
Mixed valvulopathy	18 (8, 7)
Aortic insufficiency	4 (1, 9)
Left main artery disease	7 (3.4)
Surgery	
AVR (Aortic Valve Replacement)	137 (66.8)
Full sternotomy (Isolated AVR)	28 (21,5% of isolated AVR)
Mini sternotomy (Isolated AVR)	109 (79,5% of isolated AVR)
AVR + CABG (Coronary Artery Bypass Grafting)	63 (30.7)
Other **(2 AVR + Mitral Valve Repair, 2 AVR + CABG + Mitral Valve Repair, 1 AVR + Mitral Valve Replacement)**	5 (2.4)
Total procedural time min, mean (SD)	186.9 (77.9)
CPB (Cardiopulmonary Bypass) time min, mean (SD)	108.3 (63.5)
CPB time isolated AVR time min, mean (SD)	59.1 (15.3
Ischemia time min, mean (SD)	68.2 (61.5)
Ischemia time for isolated AVR time min, mean (SD)	49.1(13.4)
Lowest temperature, mean (SD)	32.7 (4.2)
Cell SAVER, mean (SD)	627.3 (259.6)
Mean Follow-up years (SD)	6.7 (2.3–9.4)

Patients’ median BSA according to valve size is presented in Table 2. It was found that BSA differed significantly among valve sizes, in a way that patients with greater BSA had greater valve size. Moreover, BSA was significantly positively associated with valve size (rho = 0.33; *p* < 0.001).

**Table 2:** Patients’ BSA according to valve size

Valve size	BSA
	Median (IQR)
Small	1.67 (1.62 ─ 1.80)
Medium	1.73 (1.59 ─ 1.85)
Large	1.81 (1.72 ─ 1.92)
Extra large	1.91 (1.73 ─ 2.01)
P Kruskal-Wallis test	< 0.001

**Fig. 1:**
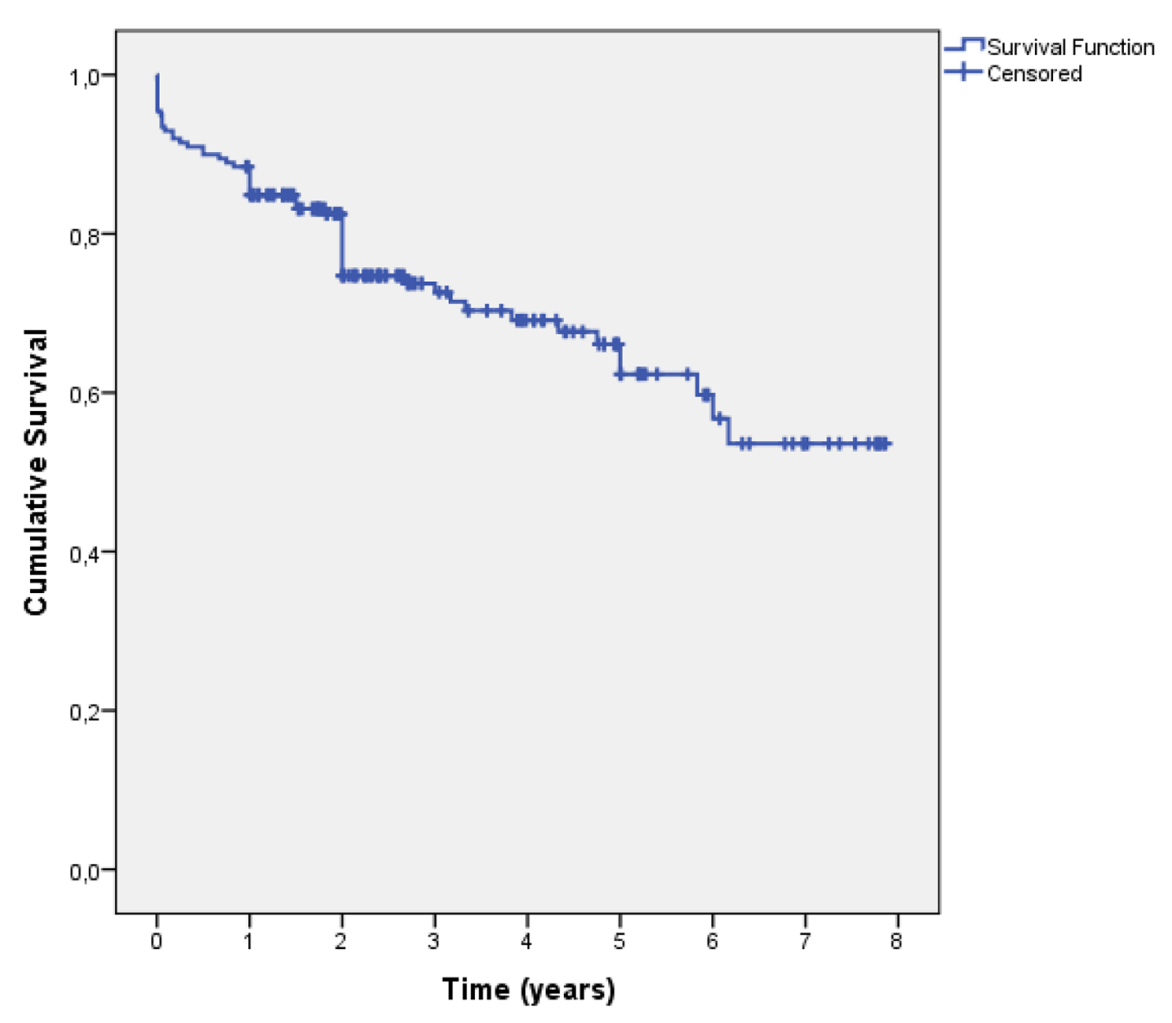
Kaplan-Meier survival curve

In total 63 (30.7%) patients died. Mean survival time was 5.5 years (SE = 0.26). Patients’ survival curve according to Kaplan-Meier method is presented in Fig. 1. Permanent pacemaker placement was 6,8% (14/205 patients), 1 reintervention due to significant paravalvular leakage, 1 case of structural valvular degeneration treated with TAVR and 3 cases of patient-prosthesis mismatch. Permanent pacemaker placement was performed in 9 patients before 2017 (9 out of 99 patients, 9,1%) and 5 patients after 2017 (5/106, 4.7%) with no statistically significant difference between these groups(*p* = 0.248). The probability of surviving at 6 months was 91.0% (SE = 2.0%), at one year 88.4% (SE = 2.3%) and at two years 82.1% (SE = 2.8%). Furthermore, 3-year surviving rate was 72.5% (SE = 3.6%) and 5-year rate was 64.8% (SE = 4.4%). The Kaplan – Meier survival curve for the total number of patients receiving sutureless aortic valves from 2013 to 2020 is presented in Fig. 1.

From univariate Cox regression analysis emerged that patients who underwent AVR + CABG had 1.82 times significantly greater hazard in comparison to patients who underwent isolated AVR (Table 3). Also, increased total time was significantly associated with greater hazard. Multivariate Cox regression analysis shown that only total time was significantly associated with surviving in a similar to the univariate analysis way (OR = 1.04; 95% CI: 1.01–1.07; *p* = 0.026). Although, the type of surgery affects the homogeneity of the sample, this study focuses on the presentation of “real-world” outcomes.

**Table 3:** Percentages of surviving and results of univariate Cox regression analysis

	Alive *N* = 142; 69.3%	Dead *N* = 63; 30.7%	HR (95% CI)+	*P*
	N (%)	N (%)		
Sex				
Men	47 (67.1)	23 (32.9)		
Women	95 (70.4)	40 (29.6)	0.81 (0.47–1.39)	0.436
Age, mean (SD)	76.6 (5.5)	75.9 (7.1)	0.98 (0.94–1.02)	0.354
BSA, median (IQR)	1.76 (1.64 ─ 1.92)	1.78 (1.67 ─ 1.91)	1.66 (0.46–6.05)	0.440
EF%, mean (SD)	52.5 (10.4)	50.7 (10.2)	0.98 (0.96–1.00)	0.105
Valve size				
Small	20 (69.0)	9 (31.0)		
Medium	59 (70.2)	25 (29.8)	0.99 (0.43–2.30)	0.981
Large	40 (67.8)	19 (32.2)	0.90 (0.36–2.21)	0.813
Extra large	23 (69.7)	10 (30.3)	1.15 (0.44–3.02)	0.777
Disease				
Aortic valve stenosis				
No	11 (55.0)	9 (45.0)		
Yes	131 (70.8)	54 (29.2)	0.65 (0.31–1.38)	0.265
Coronary artery disease				
No	103 (72.0)	40 (28.0)		
Yes	39 (62.9)	23 (37.1)	1.70 (0.99–2.94)	0.055
Mixed valvulopathy / insufficiency				
No	130 (71.0)	53 (29.0)		
Yes	12 (54.5)	10 (45.5)	1.67 (0.82–3.40)	0.158
Left main artery disease				
No	137 (69.2)	61 (30.8)		
Yes	5 (71.4)	2 (28.6)	1.37 (0.33–5.65)	0.665
Surgery				
AVR	99 (72.3)	38 (27.7)		
AVR + CABG	39 (61.9)	24 (38.1)	1.82 (1.05–3.13)	0.032
Other	4 (80.0)	1 (20.0)	1.05 (0.14–7.70)	0.962
Total procedural time min, mean (SD)	184.6 (79)	192.3 (75.5)	1.04 (1.01–1.07)	0.026
CPB time min, mean (SD)	107.6 (68.6)	110 (50.6)	1.01 (0.97–1.05)	0.553
Ischemia time min, mean (SD)	64.2 (39.2)	77.3 (94.0)	1.02 (0.99–1.04)	0.205
Lowest temperature	32,7 (4, 9)	32,6 (1, 7)	0.99 (0.91–1.07)	0.747
Cell SAVER	621,4 (255,8)	640,3 (269,5)	1.05 (0.95–0.15)	0.355

+Hazard Ratio (95% Confidence Interval)

In addition to this, deaths are divided and adjusted to three main categories of causative factors and the findings are presented in Table 4. (Table 4) The results suggest that one in five (19%) patients died from causes not related to cardiac function, while one in four patients (25.4%) or 7,8% of the total number of study population died within 3 months from the surgery. A detrimental cardiac event (syncope, arrythmia, myocardial ischemia etc.) leading to death was the probable cause of death in 35 patients (55.6%). It should be mentioned that 14 out of 16 early period deaths happened before 2017, while only 2 patients died within 3 months after surgery after 2017.

**Table 4:** Deaths adjusted to causative factors

	N	%
Death related to cardiac event	35	55.6
Death during the early postoperative period (3 months)	16	25.4
Death not related to cardiac event	12	19.0

The data were further processed in time to investigate the characteristics of the involved patients. More specifically, the severity of the patients submitted to surgery based on the preoperative Euroscore II was calculated for all of them in chronological order. This evidence indicate that the severity of the cases was higher for the years 2014, 2015, 2016 and 2017 (mean value of Euroscore II: 4.145, 3.950, 5.550 and 3980 respectively). On the other hand, the mean values of the Euroscore II are lower for the years 2018, 2019 and 2020 (mean value of Euroscore II: 3.475, 3.725 and 3.390). The mean value Euroscore II for 2013 was the lowest 3.280 but any analysis isn’t reliable due to very low number of patients.

**Fig. 2:**
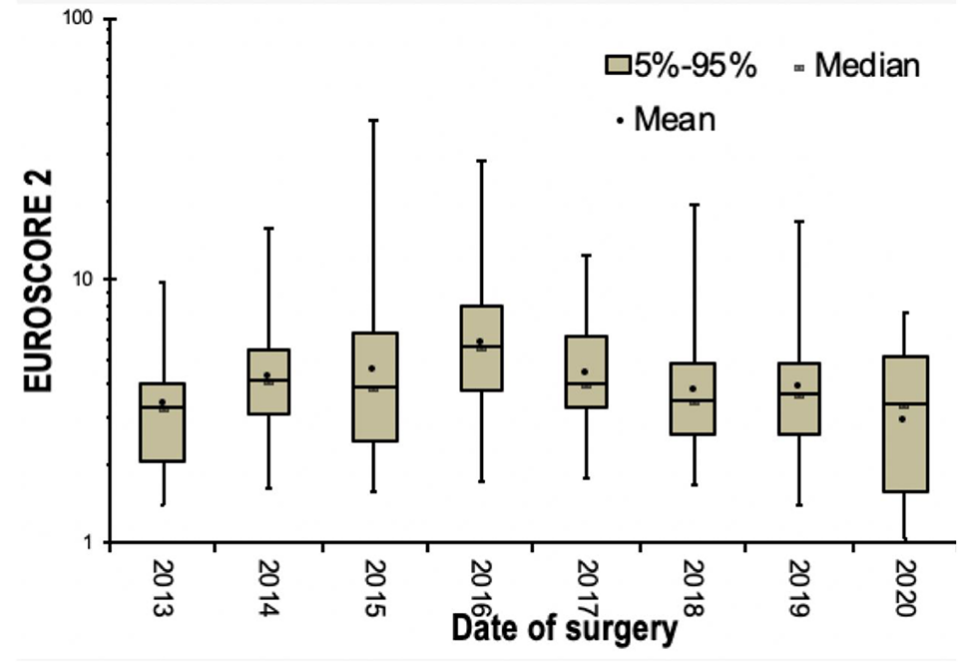
Boxplot depicting Euroscore II in groups of calendar years

The aim of this analysis is to highlight the fluctuation of the Euroscore II during the study period and mostly in relation with the initiation of the TAVR program in Greece. More specifically, the TAVR program was initiated in mid-2017 in our hospital (15/6/2017) and this factor is crucial in the evaluation of sutureless aortic valves assessment. From 2013 to 2017, high risk patients were submitted to surgical aortic valve replacement in the absence of the choice of transcatheter intervention. After the TAVR program initiation in 2017, a not negligible number of very high-risk patients were treated with percutaneous interventions. In Fig. 2, this change is depicted in a boxplot diagram. (Fig. 2) The time of TAVR program approval coincides with the decrease of mean value of Euroscore II. It can be easily assumed that since the TAVR approval, a percentage of very high-risk patients was treated with TAVR instead of surgery. Consequently, in the following years after 2017, the severity of the patients based on Euroscore II is lower and its impact on the long-term clinical outcomes is anticipated with great interest.

## Discussion

This study includes all the patients submitted to aortic valve replacement with the use of sutureless bioprosthesis in a high-volume center within a decade. The total number is large enough (205 cases) and comparable with other significant studies and trials on this field [2, 3, 7, 8]. The characteristics of the participants in this study were similar to the published data regarding the usage of Perceval-S as the mean age was 76.4 years, there was a female predominance detected (65.9%) and the mean BSA was 1.76 (1.66–1.91) [9, 10, 11]. In this study, the operative parameters measured were also similar to the existing literature [7-11]. These findings suggest that this bioprosthesis is a well-accepted and commonly used option worldwide in certain groups of patients. Although the simplification can be misleading, the typical patient, in whom Perceval-S is implanted, is approximately 75–77 years old, high-risk patient, mostly female, with small aortic diameter. Moreover, the profile of patient described is in need of the lesser feasible procedural duration due to serious comorbidities, a goal that is achieved with the implementation of the sutureless bioprosthesis in comparison with other aortic valves [12]. In fact, the multivariate Cox regression analysis in this population also showed that time does matter, as total procedural time was significantly associated with surviving. At this point, it should be mentioned that CPB and ischemia time are, in general, associated with survival but in this study, this fact wasn’t confirmed. However, total procedural time, including CPB time, ischemia time, preparation to CPB and/or harvesting time, was found so, indicating that overall extent of intervention is a determinant of the outcome. Moreover, this finding is consisted with the detection of 1.82 times significantly greater hazard of patients submitted to AVR + CABG in comparison to those submitted to AVR.

In this study, the one-year survival rate was 88,4% and the 5-year was 64,8%., which is lower compared to the findings of the recent systematic review of Williams et al. (94,9% and 84,2% respectively) [9]. In the assessment of these the data, it should be emphasized that the study can be divided in two periods. The early period from 2013 to 2017, in which the outcomes are worst, and the more recent period which begins after 2017. The early period there were two main factors contributing to lower efficiency, the lower level of experience and the lack of transcatheter techniques for aortic valve replacement. Therefore, during this period there were patients with almost prohibitive risk for surgery, who were treated surgically in the absence of other options and in the same time, Perceval-S was the preferred aortic valve in this high-risk group due to its benefits. TAVR program initiation changed the proportion of patients treated with sutureless bioprosthesis, based on mean Euroscore II, and subsequently a shift towards lower severity cases was detected for Perceval-S. In other words, patients with extremely high operative risk were treated with TAVR, while patients with lower risk compared to them but still of high operative risk, sometimes in combination with other lesions requiring intervention, were submitted to Perceval-S implantation. Hence, Perceval-S was the main choice for patients of the “grey zone” between conventional surgical valves and TAVR. The patients’ characteristics and preoperative clinical condition are undeniably factors that affect the outcomes and in a logically optimistic view the results are going to improve. Additionally, both European and American guidelines on valvular disease tend to expand the indications for TAVR, especially for patients older than 75 years [13, 14]. This factor may also contribute to further changes in the proportion of patients treated with Perceval-S, as the mean age in this cohort was 76.4 years old. In this thought, we expect that the outcomes as regards the 5-year survival rate may improve in time, but this is just a hypothesis that needs to verified in the near future.

Additionally, when assessing these outcomes, two key points should be also highlighted. After processing the group of the dead patients, an interesting finding is that 25,4% of the patients died within three months from the surgery. The 30-day mortality rate was 4,4% which is elevated in comparison to other reported outcomes ranging from 0–2,5% [3, 9, 10, 11, 15], which is mostly attributed to the early period of Perceval-S usage. This incidence reveals that a significant percentage of high-risk patients didn’t recover from the surgery at all, and this finding is related to the medical status of the patients before the procedure. Perceval-S was chosen due to its benefits for high-risk patients marginally eligible for surgery in whom all the other surgical approaches probably would be less effective. This fact is depicted by the proportion of early deaths before and after 2017. More specifically, after TAVR initiation 2 patients died within 3 months after surgery while 14 patients died in the period before 2017 respectively. As extremely high-risk patients were submitted to TAVR, lower severity cases were treated with Perceval-S which impacted positively in early period mortality. On the other hand, it should be mentioned that one in five (19%) patients died from non-related to cardiac function cause, mostly different types of cancer. After taking under consideration these findings, the adjusted mortality probably related to cardiac event after the postoperative period is decreasing to 35/205 patients, which is satisfactory enough outcome for high-risk patients. In addition to this new evidence show that Perceval-S is associated with satisfactory clinical outcomes and hemodynamic performance, while freedom from re-intervention reaches 97,6% [16].

The findings of this study suggest that CPB time, ischemia time and total procedural time were shorter in comparison to conventional aortic valve replacement due to sutureless bioprosthesis implementation, while mini sternotomy was used in about 4 in 5 patients [17]. Perceval-S has the advantage of avoiding suturing in small and calcified aortic annulus and thus reducing the duration of surgery which impacts on the operative risk [18]. In addition to this, the technique of sutureless bioprosthesis placement facilitates minimal invasive approaches with less surgical trauma and its benefits [19].

## Conclusions

Perceval-S has become a reliable and commonly used option in surgical aortic valve replacement since its first implantation in human 15 years before. In the long term follow up, the outcomes as regards the survival rate are satisfactory enough in high-risk patients. Valve dysfunction was limited in few cases. Mini sternotomy was the main surgical incision in isolated AVR favoring less invasive approaches. The proportion of patients treated with Perceval-S has changed since TAVR initiation and in the following years its efficacy on “grey zone” patients is anticipated with great interest.

## Data Availability

The datasets used and/or analyzed during the current study are available from the corresponding author on reasonable request.

## Abbreviations

TAVR: Transcatheter Aortic Valve Repair
